# A survey of knowledge, attitudes and practices towards avian influenza in an adult population of Italy

**DOI:** 10.1186/1471-2334-8-36

**Published:** 2008-03-17

**Authors:** Gabriella Di Giuseppe, Rossella Abbate, Luciana Albano, Paolo Marinelli, Italo F Angelillo

**Affiliations:** 1 Department of Public, Clinical and Preventive Medicine, Second University of Naples, Naples, Italy

## Abstract

**Background:**

Several public health strategic interventions are required for effective prevention and control of avian influenza (AI) and it is necessary to create a communication plan to keep families adequately informed on how to avoid or reduce exposure. This investigation determined the knowledge, attitudes, and behaviors relating to AI among an adult population in Italy.

**Methods:**

From December 2005 to February 2006 a random sample of 1020 adults received a questionnaire about socio-demographic characteristics, knowledge of transmission and prevention about AI, attitudes towards AI, behaviors regarding use of preventive measures and food-handling practices, and sources of information about AI.

**Results:**

A response rate of 67% was achieved. Those in higher socioeconomic classes were more likely to identify the modes of transmission and the animals' vehicles for AI. Those older, who knew the modes of transmission and the animals' vehicles for AI, and who still need information, were more likely to know that washing hands soap before and after touching raw poultry meat and using gloves is recommended to avoid spreading of AI through food. The risk of being infected was significantly higher in those from lower socioeconomic classes, if they did not know the definition of AI, if they knew that AI could be transmitted by eating and touching raw eggs and poultry foods, and if they did not need information. Compliance with the hygienic practices during handling of raw poultry meat was more likely in those who perceived to be at higher risk, who knew the hygienic practices, who knew the modes of transmission and the animals' vehicles for AI, and who received information from health professionals and scientific journals.

**Conclusion:**

Respondents demonstrate no detailed understanding of AI, a greater perceived risk, and a lower compliance with precautions behaviors and health educational strategies are strongly needed.

## Background

The first known direct avian to human transmission of influenza A (subtype H5N1) viruses was reported during an outbreak in Hong Kong in 1997 and exposure to infected poultry was identified as the probable route of transmission [1-3].

Since then, outbreaks of the H5N1 highly pathogenic avian influenza strain have been identified in birds, wild and domestic poultry, in several countries, particularly in Vietnam, Indonesia, Thailand, China, Cambodia and, more recently, in Turkey and Iraq. In Italy, no human cases have been reported and two epidemics occurred in poultry causes by avian influenza virus H5 and H7 subtypes. Alongside these massive avian outbreaks, the World Health Organization (WHO) reported more than 300 confirmed human cases of avian influenza A (H5N1), approximately two thirds of whom have subsequently died [4]. Nearly all of these cases are traceable to exposure to infected poultry or birds, but there has not yet been a mutation allowing the H5N1 and H7N7 viruses to spread efficiently in human [5].

However, concern is widespread that the current situation favors the emergence of a highly pathogenic influenza virus with the ability for efficient transmission from person to person, particularly in the presence of a mutation in the viral genome leading eventually another pandemic human influenza.

Several public health strategic interventions are required for effective disease prevention and control of the multifaceted issues posed by avian influenza [6]. Of these interventions, it is necessary to create a communication plan to keep the population fully and adequately informed on how to avoid or reduce exposure. A similar approach has been applied during the SARS epidemic [7] and the implementation of appropriate infection control measures was a key aspect for its control. In the past years a limited number of studies have been published investigating knowledge, attitudes, and practice about avian influenza among target groups [8,9] and general population [10-13]. This area of investigation seems to be an important one because members of the public often misinterpret their risk of health problems. Therefore, the objectives of the present investigation were to assess the knowledge, attitudes, and behaviors relating to avian influenza and to evaluate the effect of several potential predictors on such outcomes of interest in an adult population in Italy.

## Methods

This cross-sectional survey was conducted from December 2005 to February 2006 in the geographic area of Naples (Italy). A two-stage cluster sampling technique was employed to draw the required sample. In the area surveyed there were 40 schools and each school was considered a cluster. The first stage consisted of selecting four clusters through random sampling. The second stage consisted of randomly select 255 adults from the parents' files of each sampled school that contained 500 students.

The questionnaires used for this study were handed out, in sealed envelopes, to the Referent of the health education activities in each school with instructions to distribute one to each family. Families received an information sheet which explained the purpose of the project and requested that the survey be completed by one parent only, a self-administered anonymous questionnaire, and an envelope to facilitate the return of the completed questionnaire. In addition, the letter assured parents about the anonymity and confidentiality of all responses. Participants were asked to return the completed questionnaires to the school personnel anonymously via the envelope enclosed with each questionnaire. Participation was on a voluntary basis and all participants had the right to comply with or refuse participation. The response to questionnaire constituted the participants' informed consent. The study was approved by the Institutional Ethics Review Board.

The survey, that was a modification of an instrument previously used [8], was arranged in different sections enquiring about participants' demographic and socioeconomic characteristics, knowledge of the definition, modes and vehicles of transmission, risk groups, and preventive measures about avian influenza, attitudes towards avian influenza, behaviors regarding use of preventive measures and food-handling practices, whether they had ever received advice and information about avian influenza and, if so, the sources. The response choices for all knowledge questions were given on a three-point Likert-type scale using "yes", "no", "do not know" options for the modes and vehicles of transmission, and risk groups and "agree", "uncertain", and "disagree" for the measures concerning the preventive measures; whereas, the response choice for the question about the knowledge of the definition was open. The responses for all statements relating to attitudes towards avian influenza, to ascertain level of agreement or disagreement were given on a three-point Likert-type scale (from 1 to 3, 1 = agree, 2 = uncertain, 3 = disagree); in two questions respondents were also asked to use a ten-point Likert-type scale to assess perceived degree of risk for contracting avian influenza for him/her and for friends/familiars with responses ranged from 1 (low risk) to 10 (high risk). Five possible categories of responses were allowed, ranging from never to always, to measure compliance with recommendations of the WHO to avoid spread of avian influenza through food [6]. We have also asked whether the respondent and/or member(s) in the household, in the three months preceding the survey, have modified the habits in buying foods or in dietary habits for fear of contracting avian influenza.

The original version of the survey instrument underwent a pilot study among a convenient group of 50 subjects to ensure practicability, validity, and interpretation of answers. On the basis of the comments obtained, the questionnaire was revised in item, wording, and format before distribution to the study sample.

### Statistical analysis

Multivariate stepwise logistic and linear regression analyses investigated the independent contribution of potential predictors to the following primary outcomes of interest: knowledge about the main modes of transmission (animal-to-animal and animal-to-human) and the animals classified as common vehicles (poultry and birds) for avian influenza (model 1); knowledge that wash hands with soap before and after touching raw poultry meat and use of gloves is a hygienic practices to avoid spreading of the avian influenza virus through food (model 2); wash hands with soap before and after touching raw poultry meat and use of gloves (model 3); perception of risk of contraction avian influenza for him/her (model 4). For the purposes of analysis, the outcome variables originally consisting of multiple categories in the logistic analysis were collapsed into two levels. In Model 1, respondents were divided into those who knew the main modes of transmission (animal-to-animal and animal-to-human) and the animals classified as common vehicles (poultry and birds) for avian influenza versus all others; in Model 2, those who knew that wash hands with soap before and after touching raw poultry meat and use of gloves is a hygienic practices to avoid spreading of the avian influenza virus through food versus all others; in Model 3, those that wash hands with soap before and after touching raw poultry meat and use gloves versus all others. In all models the following independent explanatory variables were included: age, gender, marital status, educational level, number of children, employment status, health professionals and reading scientific journals as sources of information, and need of additional information. Others independent explanatory variables were also included in the different models: perception of risk of contraction avian influenza (models 1–3); knowledge about the modes of transmission of avian influenza (models 2 and 3); correct definition of avian influenza and know that avian influenza could be transmitted by eating and touching raw eggs and poultry foods (model 4); knowledge that wash their hands with soap before and after touching raw poultry meat and use of gloves is a hygienic practices to avoid spreading of the avian influenza virus through food (model 3). Before testing multivariable logistic regression models assessing predictors of the outcomes of interest, we examined correlations to assess collinearity among the independent variables and bivariate relations between the independent variables and the dependent variable. The criterion to be met before any independent variable was considered for entry into an initial multivariable logistic regression model was a *p*-value ≤ 0.25 obtained for each outcome variable in the univariate analysis and noncollinear with other predictors. Furthermore, the significance level for variables entering the logistic regression models was set at 0.2 and for removing from the model at 0.4. Odds ratios (ORs) and their corresponding 95% confidence intervals (CIs) were calculated in the model for the independent variables. When building linear regression model, we have first included only one possible variable at a time. Then, using the variables that were significant at *p*-value ≤ 0.25, we constructed a stepwise multivariate linear regression model and the significance level for variables entry the model was set at 0.2 and for removal at 0.4. Statistical significance level was defined as a two-tailed *p*-value ≤ 0.05. Stata version 8.1 software program was used for all statistical analyses [14].

## Results

The sample consisted of 683 individuals for a participation rate, defined as the number of completed questionnaires divided by the number of those randomly selected, of 67%. Socio-demographic characteristics of the respondents are reported in Table 1. The average age was 40.7 years, two thirds were female, almost all were married, the majority had not reached college level education, more than half is inactive or housewife, and one-third has three or more children.

**Table 1:** Socio-demographic characteristics of the study population

	*n*	*%*
*Gender*		
Female	461	67.5
Male	222	32.5
*Age group (years)*	40.7 ± 6.8 (40)*	
≤ 35	154	22.5
36–40	213	31.2
41–45	163	23.9
46–50	100	14.6
> 50	53	7.8
*Marital status*		
Married	654	95.7
Other	29	4.3
*Educational level (years)*		
No formal education	28	4.1
5–7	117	17.1
8–12	239	35
≥ 13	299	43.8
*Employment status*		
Employed	309	45.2
Unemployed/Housewife	374	54.8
*Number of children*		
1	64	9.4
2	372	54.4
≥ 3	247	36.2

* Mean ± Standard deviation (Median)

The respondents' knowledge about avian influenza is reported in Table 2. Half the survey respondents correctly defined avian influenza as a contagious infection caused by a virus that can affect all species of birds, and 20.1% to 81.4% knew the different modes of transmission, although 7.5% indicated a human-to-human. Almost all (95.7%) and three-quarters (74.7%) identified poultry and birds as common vehicles for the disease. Overall, only 33.5% correctly identified the modes of transmission and the common vehicles for avian influenza. Multiple logistic regression analysis showed that those employed (OR = 1.34; 95% CI 1.05–1.7) and with higher educational level (OR = 1.31; 95% CI 1.1–1.63) were significantly more likely to correctly answer knowledge questions about transmission (Model 1 in Table 3). Respondents did not recognize the major risk groups, since a large percentage agreed that poultry workers (88%) were at risk, but lower values were reported for butchers (55.1%), hunters (30.7%), and veterinarians (23.6%). Moreover, 34.6% knew that washing their hands with soap before and after touching raw poultry meat and using gloves is a hygienic practice to avoid spreading of the avian influenza virus through food. Those older (OR = 1.03; 95% CI 1.01–1.05), who knew the modes of transmission and the common vehicles for avian influenza (OR = 1.63; 95% CI 1.11–2.39), and who still need additional information about avian influenza (OR = 1.52; 95% CI 1.09–2.12) were more likely to know this practice (Model 2 in Table 3).

**Table 2:** Knowledge about avian influenza of the study population

	Yes		No		Do not know	
	*n*	*%*	*n*	*%*	*n*	*%*

***Definition *^a ^**(contagious infection caused by virus that can affect all species of birds)	351	52.5	318	47.5	0	-
						
***Modes of transmission***						
Animal-to-animal	421	61.6	0	-	262	38.4
Animal-to-human	469	68.7	0	-	214	31.3
No human-to-human	632	92.5	0	-	51	7.5
Eating uncooked poultry	551	80.7	75	11	57	8.3
Eating uncooked eggs	513	75.1	110	16.1	60	8.8
Touching uncooked poultry	556	81.4	74	10.8	53	7.8
Touching uncooked eggs	353	51.7	177	25.9	153	22.4
Touching uncooked frozen poultry	137	20.1	28	4.1	518	75.8
						
***Vehicles of transmission***						
Poultry	654	95.7	0	-	29	4.3
Birds	510	74.7	0	-	173	25.3
						
***Risk groups***						
Poultry workers	601	88	0	-	82	12
Butchers	376	55.1	0	-	307	44.9
Hunters	210	30.7	0	-	473	69.3
Veterinarians	161	23.6	0	-	522	76.4

	Agree		Uncertain		Disagree	

	*n*	*%*	*n*	*%*	*n*	*%*

***Use of preventive measures***						
Preparing raw poultry and other foods using different knifes	482	70.6	123	18	78	11.4
Touching raw poultry with gloves	382	55.9	191	28	110	16.1
Wash hands with water and soap before and after preparing raw poultry	302	44.2	258	37.8	123	18
Cleaning cutting boards after preparing raw poultry	298	43.6	221	32.4	164	24
Using gloves and washing hands with soap before and after touching raw poultry meat	236	34.6	123	18	324	47.4

^a ^The number of participants responding to this question is 669

**Table 3:** Logistic (1–3) and linear (4) regression models results

Variable	OR	95% CI	*p*
Model 1. Knowledge about the main modes of transmission and the animals classified as common vehicles for avian influenza			
Log likelihood = -419.33, χ^2 ^= 32.65 (4 df), *p *< 0.0001			
Level of education	1.31	1.1–1.63	0.015
Employment status	1.34	1.05–1.7	0.023
Number of children	0.78	0.59–1.04	0.08
Age	1.02	0.99–1.04	0.12
			
Model 2. Knowledge that wash hands with soap before and after touching raw poultry meat and use of gloves is a hygienic practices to avoid spreading of the avian influenza virus through food			
Log likelihood = -425.15, χ^2 ^= 30.27 (5 df), *p *< 0.0001			
Knowledge about the modes of transmission and the animals classified as common vehicles for avian influenza	1.63	1.11–2.39	0.011
Reported interest in receiving further information on avian influenza	1.52	1.09–2.12	0.014
Age	1.03	1.01–1.05	0.041
Health professionals and scientific journals as sources of information	1.35	0.96–1.91	0.08
Employment status	1.24	0.97–1.58	0.08
			
Model 3. Wash hands with soap before and after touching raw poultry meat and use of gloves			
Log likelihood = -423.57, χ^2 ^= 68.66 (6 df), *p *< 0.00001			
Perception of risk of contraction avian influenza	1.13	1.06–1.19	< 0.0001
Knowledge that wash hands with soap before and after touching raw poultry meat and use of gloves is a hygienic practices to avoid spreading of the avian influenza virus through food	2.42	1.73–3.4	< 0.0001
Health professionals and scientific journals as sources of information	1.65	1.17–2.34	0.004
Gender	1.53	1.08–2.17	0.015
Knowledge about the modes of transmission and the animals classified as common vehicles for avian influenza	1.57	1.07–2.32	0.02
Number of children	0.81	0.62–1.06	0.12

Variable	Coeff.	t	*p*

Model 4. Perception of risk of contraction avian influenza for him/her			
F (7,661) = 15.56, *p *< 0.00001, R^2 ^= 14%, adjusted R^2 ^= 13%			
Level of education	-0.66	-4.85	< 0.0001
Reported interest in receiving further information on avian influenza	-1.07	-4.85	< 0.0001
Knowledge that avian influenza could be transmitted by eating and touching uncooked poultry and eggs	1.07	3.09	0.002
Correct definition of avian influenza	-0.64	-3.06	0.002
Employment status	-0.40	-2.36	0.019
Age	-0.02	-1.09	0.28
Knowledge about the modes of transmission and the animals classified as common vehicles for avian influenza	-0.23	-1.01	0.31
Constant	11.12		

More than half of the respondents thought that avian influenza was a serious disease (61.9%) and that it was possible to prevent (53.3%). The respondents' level of perceived risk of contracting avian influenza for them and for friends/familiars resulted in a mean total score respectively of 5.9 ± 2.9 and 6.2 ± 2.8, indicating a high risk perception with respectively 19.3% and 20.4% of the respondents having reported feeling "very much" at risk by answering "10". A stepwise multivariate linear regression model was constructed to search for associations with the attitude, aiming at understanding which variable had stronger associations with the perception of risk of contraction avian influenza by the respondent. Respondents considered the risk for them of being infected significantly higher if they were from lower socioeconomic classes, had lower educational level, if they did not know the definition of avian influenza, if they knew that avian influenza could be transmitted by eating and touching raw eggs and poultry foods, and if they believed that they did not need additional information about the disease (Model 4 in Table 3).

Participants were asked about their behaviors regarding the use of preventive measures and food-handling practices. Approximately two-thirds reported that, in the three months preceding the survey, at least one member in the household had modified the habits in buying foods (63.3%) or in dietary (59.9%) for fear of contracting avian influenza. As regards hygienic practices to avoid spreading of the virus through food, among those who prepared food, only 26.9% reported washing their hands with soap before and after touching raw poultry meat and using gloves. Multivariable logistic regression analysis indicated that compliance with the hygienic practices was more likely by those who perceived a higher risk of contracting avian influenza (OR = 1.13; 95% CI 1.06–1.19), by those who knew that washing hands with soap before and after touching raw poultry meat and using gloves is hygienic practice to avoid spreading of the virus through food (OR = 1.57; 95% CI 1.07–2.32), by those who knew the modes of transmission and the common vehicles for avian influenza (OR = 2.42; 95% CI 1.73–3.4), and by those who received information by health professionals and scientific journals (OR = 1.65; 95% CI 1.17–2.34) (Model 3 in Table 3).

Almost all respondents recalled receiving some information about avian influenza (97.9%) mostly through mass media (85.8%), health professionals (26.5%), and scientific journals (8.4%). A vast majority (65%) reported interest in receiving further information on avian influenza.

## Discussion

The results of the present survey depict a mosaic of opinions outlining the stated knowledge, attitudes, and self-reported behavior patterns concerning avian influenza among a large cross-section of a random sample of an adult population in one region of Italy. Guidelines and recommendations have been developed to prevent and control the spread of avian influenza at source and in responding to the pandemic threat [6,15-19]. These attempts to provide public health related measures in the community, in workers involved in outbreak disease control and eradication activities, in people involved in producing, marketing, and living with poultry, and in travellers who are visiting countries experiencing outbreaks. The main recommended measures which need to be used in concert, are: 1) intensify collaboration between the animal and public health sectors; 2) appropriate personal protective equipment for medical workers that transport/treat avian flu patients and for workers involved in the culling, transport or disposal of avian influenza-infected poultry; 3) effective disease surveillance for early detection and reporting of outbreaks; 4) food safety of poultry products; 5) control of movement of birds and products that may contain virus; 6) risk communication; 7) rapid, humane destruction of infected poultry and poultry at high risk of infection, and 8) proper use of vaccination.

Data gathered showed that a high number of respondents had no detailed understanding of avian influenza. Specifically, less than half answered correctly the questions on the modes and vehicles of transmission and on the recommended hygienic practices to avoid spreading of the avian influenza virus through food. Moreover, it was disturbing to note that detailed questioning revealed gaps in knowledge about the risk groups. That this happened, despite the fact that almost all received information about avian influenza from different sources, is troubling. In rural Thailand a community cluster survey on 200 people has shown widespread knowledge regarding avian influenza and the effective means of protection with, for example, 76% recognizing that people could get the disease from chicken or other poultry [11]. In our study, the investigation of correlates in multivariate comparison, which allowed us to control for different risk factors, yielded several interesting findings such as that older respondents with a higher educational level and from higher socioeconomic class were more likely to be knowledgeable. The inclusion of measures such as participants' level of schooling and employment status greatly reduces the size differences between groups. Failure to account for these socioeconomic factors would result in biased estimates and artificially high differences across groups. Finally, most respondents recognized that their knowledge on preventive measures was fair and indicated the need for increasing that knowledge. This finding is important because it has already been reported that public health education campaigns and general media reports about avian influenza appear to have been effective in reaching those who were at greatest risk of acquiring the disease through contact with backyard poultry [11].

This study revealed a relatively high degree to which respondents themselves perceived themselves to be at risk of avian influenza virus as a health threat and demonstrated a readiness on the part of respondents to be educated. In our sample respectively 19.3% and 20.4% said that they were "very much" concerned about the risk of contracting avian influenza for them and for friends/familiars in the future. In a previous telephone study, that examined the perceived risk of avian influenza from live chicken sales involving Hong Kong households, it was documented that one third of those surveyed perceived some risks and almost 50% indicated that their friends had expressed anxieties [10]. It has been well established that worried individuals were significantly more likely to have received advice or instruction about the disease by health professionals and by reading scientific journals and to feel the need for more information. Our findings do suggest that the perception of risk was a significant determinant of greater compliance with recommended precautionary procedures. Indeed, respondents worried about their own risk were more likely to use gloves and wash hands with soap before and after touching raw poultry meat. Such findings suggest that the population would both welcome and benefit from tools and strategies that would help them to reduce their fear because it is important in whether or not they adhere to these procedures.

The respondents to our questionnaire exhibited higher compliance with recommendations of the WHO to avoid spread of avian influenza through food [6], such as hand washing and using protective gloves, when compared with the findings reported in two previous surveys about food-borne diseases. In one study only 20.8% of respondents claimed that they used protective gloves, 53.9% reported washing hands before and after touching raw and unwrapped food, and 50.4% reported using soap to wash hands [20]. In the other one respectively 68.7% and 66.2% of food handlers routinely washed their hands before and after handling any food [21]. As we hypothesized, in accordance with a previous study, knowledge influences behavior [8]. Our survey indicates a significant association between those who fail to wash hands and to use gloves and the lack of knowledge that these are standard hygienic practices to avoid spreading of the virus through food. In addition, it is notable that the main source of information was the media and not qualified healthcare representatives. It has been observed that our results confirmed the hypothesis that those who received information from health professionals and scientific journals had higher relevant compliance than those who did not receive information from these sources. These findings support the importance for media campaigns to implement educational and policy strategies and to increase patient education by medical professionals in the context of routine medical care.

Despite the novelty and significance level of these findings, some methodological considerations ought to be highlighted when interpreting our results. First, the analyses were based on cross-sectional data and the findings of the analysis of factors associated with the outcomes of interest should be interpreted with caution given the nature of the associations that limited us from drawing definitive causal conclusions or direction of causality about the observed relationships between among independent characteristics and the outcomes measured. However, we feel that our study design was adequate to assess the knowledge, attitudes, and behaviors relating to avian influenza and identify what percentage of these could be explained by several potential predictors. Second, the study was limited to those parents of children in randomly selected schools, which may have implications for the generalizability of the results. However, because in our country the education is mandatory until the age of 16 irrespective of the characteristics of the parents, we believe our results are generalizable to all population. Third, all variables used in this analysis were gathered using entirely respondents' self-reports and self-perceptions, and biases in perception and reporting cannot be ruled out. The problem with self-reporting is that participants' responses may reflect intentionally or unintentionally perceived desirable responses or an attempt to inflate or minimize reports of behaviors. However, to gain a true reflection of knowledge and behavior, respondents were assured that their responses would not be shared, since the survey was delivered with the responses that were anonymous to encourage accurate recording. Fourth, it was not possible to identify characteristics of those who failed to return the questionnaire, so it was not possible to establish whether they were in any way different from those who did return it. However, there is no obvious reason to suspect that non-responders were substantially different from responders. Despite the potential limitations, the main advantage of the current study is that we were able to achieve a relatively large sample and the high response rate excludes one major potential source of bias in the results. We believe that this high response rate was made possible through the extreme importance of the topic surveyed.

## Conclusion

In conclusion, the results of this study illustrates that, despite being given information, respondents had no detailed understanding of avian influenza, had a great perceived risk of experiencing avian influenza, and had a low compliance with precautions behaviors. These observations raise concerns about a clear need to find the optimal way of correcting these deficiencies by developing and implementing public health policy regarding priorities for tailored educational and promotion strategies and in particular more attention should be given on using preventive approaches in these population. Nevertheless, it is important to consider that dissemination and widespread adoption of preventive measures require education. Encouragingly, respondent's interest in learning more about avian influenza was high in our survey. Therefore, designing and implementing avian influenza educational programs and measuring their effectiveness should be priorities to incentive the population to take a more active role.

## Competing interests

The author(s) declare that they have no competing interests.

## Authors' contributions

GDG and RA participated in the design of the study, data collection, statistical analysis and interpretation of the data. PM participated in the design of the study and interpretation of the data. LA participated in the statistical analysis. IFA, the principal investigator, designed the study, was responsible for the data collection, statistical analysis and interpretation, and wrote the article. All Authors read and approved the final manuscript.

## Pre-publication history

The pre-publication history for this paper can be accessed here:


